# PI3K/AKT/mTOR Signaling Regulates the Virus/Host Cell Crosstalk in HPV-Positive Cervical Cancer Cells

**DOI:** 10.3390/ijms20092188

**Published:** 2019-05-03

**Authors:** Felicitas Bossler, Karin Hoppe-Seyler, Felix Hoppe-Seyler

**Affiliations:** Molecular Therapy of Virus-Associated Cancers (F065), German Cancer Research Center (DKFZ), D-69120 Heidelberg, Germany

**Keywords:** human papillomavirus, tumor virus, cervical cancer, head and neck cancer, hypoxia, AKT, PI3K, mTOR, senescence, immunotherapy

## Abstract

Human papillomavirus (HPV)-induced cancers will remain a significant clinical challenge for decades. Thus, the development of novel treatment strategies is urgently required, which should benefit from improving our understanding of the mechanisms of HPV-induced cell transformation. This should also include analyses of hypoxic tumor cells, which represent a major problem for cancer therapy. Recent evidence indicates that the PI3K/AKT/mTOR network plays a key role for the virus/host cell crosstalk in both normoxic and hypoxic HPV-positive cancer cells. In normoxic cells, the efficacy of the senescence induction upon experimental E6/E7 repression depends on active mTORC1 signaling. Under hypoxia, however, HPV-positive cancer cells can evade senescence due to hypoxic impairment of mTORC1 signaling, albeit the cells strongly downregulate E6/E7. Hypoxic repression of E6/E7 is mediated by the AKT kinase, which is activated under hypoxia by its canonical upstream regulators mTORC2 and PI3K. This review highlights our current knowledge about the oxygen-dependent crosstalk of the PI3K/AKT/mTOR signaling circuit with the HPV oncogenes and the phenotypic state of the host cell. Moreover, since the PI3K/AKT/mTOR pathway is considered to be a promising target for anticancer therapy, we discuss clinical implications for the treatment of HPV-positive cervical and head and neck squamous cell carcinomas.

## 1. Introduction

Human papillomaviruses (HPVs) are major risk factors for the development of oropharyngeal and anogenital cancers. Especially cervical cancer is well characterized for being caused by a persistent infection with oncogenic, so-called “high-risk” HPV types [1,2]. The transforming abilities of high-risk HPVs are largely attributed to the viral E6 and E7 oncoproteins, which exert pleiotropic functions [3,4,5]. The tumor suppressor proteins p53 and pRb are critical targets that are inactivated by E6 and E7, respectively. Ultimately, E6 and E7 deregulate cell proliferation, lead to genomic instability and subvert important anti-tumorigenic pathways, such as the induction of senescence or apoptosis, eventually leading to cancer [1,3,4,5].

With the development of effective prophylactic vaccines based on the HPV major capsid protein L1, a major achievement in the prevention of cervical cancer was accomplished [6,7]. These vaccines are protecting against infections with the most common oncogenic HPV types. However, they cannot prevent cancer development in already persistently infected individuals [8,9], a process that typically occurs over decades [1,2]. Moreover, since worldwide vaccination rates are low, especially in low-income countries where incidence rates are high [10], cervical cancer continues to pose an important clinical challenge with an urgent need for new and improved therapeutic approaches. A better understanding of HPV-associated carcinogenesis will be crucial for the future design of novel treatment strategies.

Many cancers possess hypoxic subregions (i.e., containing oxygen concentrations below 1.5% [11]). Hypoxia is a negative prognostic factor that is associated with immunosuppression [12,13], increased resistance towards chemo- and radiotherapy and poor patient prognosis in many cancers, including those linked to HPV infections [14,15,16,17]. The regulatory principles underlying the crosstalk between the HPV oncogenes and their host cell under hypoxia are thus far only incompletely understood [18]. Recently, it was uncovered that hypoxic cervical cancer cells can strongly downregulate E6/E7 expression and enter a reversible proliferative arrest [19]. This dormant state could contribute to the immune evasion and therapy resistance of hypoxic HPV-positive cancer cells (for a more detailed discussion, please refer to refs. [4,18,19]). Further, since -upon reoxygenation- dormant HPV-positive cancer cells can reactivate E6/E7 expression and resume proliferation, this cell population may serve as a reservoir for tumor recurrence after treatment [19]. Collectively, these findings show that, in order to improve the treatment outcome, it is of great importance to develop therapeutic strategies that are also effective against hypoxic cancer cells.

Several proteins and pathways have been implicated in orchestrating the cellular response to hypoxia. A central role is played by the hypoxia-inducible factors (HIFs), which are transcription factors serving as master regulators of the cellular adaptation to hypoxia by modulating the expression of a plethora of genes [20]. Another important hypoxia-responsive factor is the AKT kinase [21,22,23]. AKT is a central member of the phosphoinositide 3-kinase (PI3K)/mechanistic target of rapamycin (mTOR)/AKT signaling network [24]. Recent findings reveal that there is a complex interplay between the PI3K/AKT/mTOR signaling cascade, the regulation of HPV oncogene expression and the phenotypic response of HPV-positive cancer cells to E6/E7 repression, which all are dependent on the cellular metabolic and oxygenation status [4,18,19,25]. This review summarizes the most recent findings in this context and discusses the implications thereof.

## 2. The PI3K/AKT/mTOR Pathway and Cancer

**Figure 1:**
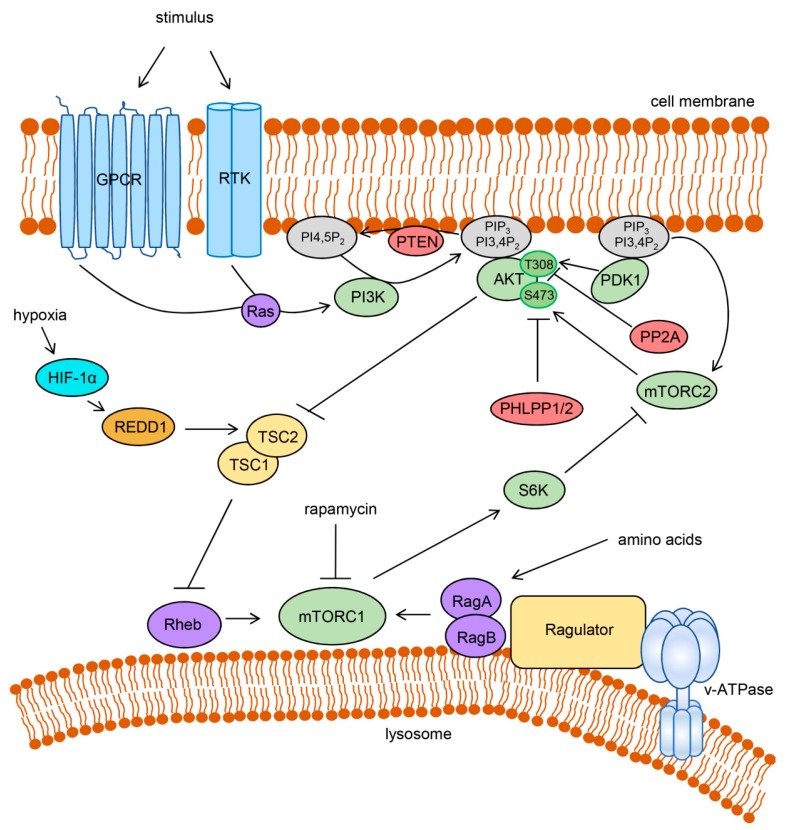
Activation and regulation of the PI3K/AKT/mTOR signaling network. Extracellular stimuli activate PI3K through GPCRs or RTKs, often involving Ras GTPases. Active PI3K produces PIP_3_ and PI3,4P_2_ that recruit AKT to the cell membrane. Phosphoinositide-dependent PDK1 and mTORC2 activate AKT by phosphorylation. AKT activation is negatively regulated by direct dephosphorylation through PP2A and PHLPP1/2 and by dephosphorylation of phosphoinositides through phosphatase and tensin homolog (PTEN). AKT can activate mTORC1 by inhibiting tuberous sclerosis complex 2 (TSC2), which in complex with TSC1, acts as a GTPase activating protein (GAP) for Rheb. Rheb as well as the RagA:RagB heterodimer is required for the activation of mTORC1. The amino acid-dependent RagA:RagB interacts with the lysosome-associated protein complex Ragulator that is bound to the lysosomal v-ATPase. mTORC1 activity can be inhibited under hypoxia by HIF-1α-mediated transcriptional stimulation of REDD1 (regulated in development and DNA damage response 1) which activates TSC2. mTORC1 can orchestrate a negative feedback regulation, for instance by inhibition of mTORC2 through the activation of S6K. Rapamycin directly inhibits mTORC1 but not mTORC2. Arrows indicate an activation, bar-headed lines indicate an inhibition.

The PI3K/AKT/mTOR signaling cascade influences many fundamental aspects of cellular biology by promoting cell survival, growth, proliferation, migration and energy metabolism. Aberrant activation of this pathway can contribute to the malignant phenotype of tumor cells and to therapy resistance. As a consequence novel therapeutic agents targeting this pathway are under development and in clinical tests [26,27,28]. Extensive research in the last years provided a better understanding of the mechanistics of this complex network (Figure 1) that centers on the serine/threonine kinase AKT, which comprises three distinct isoforms (AKT1-3) [24].

For the stimulation of AKT signaling, receptor tyrosine kinases (RTKs) or G protein-coupled receptors (GPCRs) are activated by extracellular stimuli such as growth factors (e.g., insulin) or cytokines, resulting in the recruitment and activation of the lipid kinase PI3K, often through RAS GTPases [24] (Figure 1). Active PI3K phosphorylates the membrane lipid phosphatidylinositol-4,5-bisphosphate (PI4,5P_2_) to generate phosphatidylinositol-3,4,5-trisphosphate (PIP_3_) that can be further converted to phosphatidylinositol-3,4-bisphosphate (PI3,4P_2_). AKT can then be recruited to the cell membrane by the interaction of its N-terminal pleckstrin homology (PH) domain with PIP_3_ or PI3,4P_2_. This allows phosphorylation of the T308 residue of AKT1 (T309 and T305 for AKT2 and AKT3, respectively) by phosphoinositide-dependent kinase-1 (PDK1) that is itself recruited to the membrane by binding to phosphoinositides through its PH domain [24]. Phosphorylation of AKT1 at a second residue, S473 (S474 and S472 for AKT2 and AKT3, respectively), is required for full enzymatic activity [29]. This phosphorylation is carried out by mTOR complex 2 (mTORC2), one of the two distinct protein complexes that contain the mTOR kinase [30]. Upstream in this cascade, the activity of mTORC2, like PDK1, is dependent on the growth factor signaling-induced activation of PI3K [31,32]. Functionally, mTORC2 is largely insensitive to rapamycin, which is an efficient inhibitor of mTOR complex 1 (mTORC1) signaling [33].

Activated AKT regulates a large number of downstream targets, amongst those mTORC1, which is activated by AKT primarily by the inhibition of tuberous sclerosis complex 2 (TSC2) (Figure 1). TSC2 forms a complex with TSC1 and acts as a GTPase-activating protein (GAP) for the GTPase Rheb. Upon being phosphorylated by AKT, TSC2 no longer reduces the amount of GTP-bound Rheb, which can then bind and activate mTORC1 [24]. Hence, similar to mTORC2, mTORC1 requires PI3K-mediated growth factor signaling for its activation. However, mTORC1 is additionally dependent on the nutrient status of the cell. Only in the presence of sufficient amino acids, Rag GTPases promote recruitment of mTORC1 to the cytoplasmic face of the lysosome, where they interact with a protein complex called Regulator that itself interacts with the lysosomal v-ATPase. Here, mTORC1 can interact with Rheb [34,35]. Since mTORC1 induces cell growth and strongly promotes anabolic processes such as protein, lipid and nucleotide synthesis, this regulatory mechanism ensures that these energy- and building block-consuming processes are not carried out under nutrient-depleted conditions [35]. In line with this, mTORC1 is also responsive to the cellular energy and oxygen status [36].

Negative regulation of PI3K-mediated activation of AKT is carried out by the phosphoinositide phosphatase PTEN (phosphatase and tensin homolog) that reverses the generation of PIP_3_ or by the protein phosphatases PP2A and PHLPP1/PHLPP2 that dephosphorylate AKT (Figure 1). Additionally, AKT signaling is regulated by feedback inhibition induced by some of its downstream targets. In this context, several mechanisms of mTORC1-induced inhibition of AKT activation have been described. For instance, mTORC1 enhances the degradation of the insulin receptor substrates 1 and 2 (IRS1, IRS2) and stabilizes the growth factor receptor bound protein 10 (GRB10) resulting in limited PI3K activation by RTKs such as the insulin and insulin-like growth factor 1 (IGF1) receptors. Moreover, through the activation of S6 kinase (S6K), mTORC1 inhibits mTORC2 and diminishes mTORC2-mediated phosphorylation of AKT [24].

Overall, the PI3K/AKT/mTOR cascade expands to a broad and complex signaling network which coordinates the response of the cell to multiple internal and external stimuli, thereby affecting key processes linked to carcinogenesis, such as cell metabolism, growth, proliferation and survival [24,35].

## 3. mTOR Signaling and Senescence in HPV-Positive Cancer Cells

HPV-positive cancer cells are considered “oncogene-addicted” since they are dependent on the continuous expression of the two viral oncogenes E6 and E7 to maintain their malignant phenotype [1]. This is supported by studies showing that the inhibition of E6/E7 expression in HPV-positive cancer cells rapidly leads to cellular senescence [37,38,39], a largely irreversible proliferative arrest that can be induced in response to abnormal proliferative stimuli (e.g., activated oncogenes) in order to prevent the outgrowth of deregulated cells [40,41]. In E6/E7-transformed cells, the viral oncogenes actively suppress senescence largely by inhibiting the p53 and pRb pathways [42,43]. Upon repression of E6/E7, for instance through RNA interference or ectopic expression of the HPV E2 protein (which blocks the E6/E7 transcriptional promoter in oncogenic HPV types), p53 and pRb are reactivated and induce further anti-proliferative effectors [38,42,43]. Notably, the induction of senescence upon E6/E7 repression is impaired in hypoxic HPV-positive cancer cells [19], as further detailed below.

In addition to the activation of p53- and pRb-linked pathways, efficient senescence induction in HPV-positive cancer cells upon E6/E7 repression requires the activity of mTORC1 [19], which has a growth promoting potential. This finding is in accordance with a model proposed by Blagosklonny et al. stating that senescence is the result of a cell cycle arrest that converts into an irreversible arrest due to the presence of conflicting growth-stimulatory signaling, e.g., via mTORC1, a process termed geroconversion [44]. Senescent cells characteristically maintain a high metabolic rate and develop an enlarged morphology [40], which is consistent with the functions of mTORC1 in anabolic processes that promote cell growth [35]. Another key feature of many senescent cells is the senescence-associated secretory phenotype (SASP); the secretion of pro-inflammatory mediators like growth factors, cytokines or proteases [40]. This phenotype can also be regulated by mTORC1 [45,46]. Since senescent cells usually permanently exit from the cell cycle, senescence represents a protective mechanism against tumor development and can be a desired outcome of cancer therapy [47]. However, it becomes increasingly clear that senescence also has adverse pro-tumorigenic effects since secreted SASP components can contribute to tumor progression by promoting cell proliferation, inflammation, angiogenesis and metastasis [48].

Efficient senescence induction in HPV-positive cancer cells upon E6/E7 repression, as well as through other pro-senescent stimuli (e.g., chemotherapy), depends on the activity of mTORC1 as it can be counteracted when cells are treated with rapamycin [19,49]. The major role for mTORC1 in the induction of senescence upon E6/E7 repression is further corroborated by findings obtained under hypoxic conditions: Hypoxic HPV-positive cancer cells strongly downregulate E6/E7, yet they enter a reversible growth arrest and retain their proliferative potential [19]. This phenotypic response is linked to the hypoxia-mediated downregulation of mTORC1 signaling that prevents senescence-inducing geroconversion [19,50].

Hypoxia has been described to repress mTORC1 signaling by various mechanisms, e.g., by the inhibition of the interaction of mTORC1 with its activator Rheb [51,52] or by the activation of the negative regulator TSC1/TSC2 [53,54,55,56]. In hypoxic HPV-positive cancer cells, silencing of REDD1 (regulated in development and DNA damage response 1) or TSC2 expression restores phosphorylation of mTORC1 targets and substantially increases the fraction of senescent cells [19]. REDD1 is a hypoxia-induced HIF-1α target gene that has been shown to enable dissociation of TSC2 from 14-3-3 and thus, allows TSC2 to inhibit mTORC1 activity under hypoxia [53,57]. Hence, the REDD1/TSC2 axis is, at least partially, responsible for the repression of mTORC1 signaling in hypoxic HPV-positive cancer cells. Interestingly, mTORC1 is repressed in hypoxic HPV-positive cancer cells despite an induction of its upstream activator AKT [25], suggesting that the hypoxia-linked mTORC1 inhibition through REDD1/TSC2 is dominant over the AKT-mediated phosphorylation of TSC2 and subsequent mTORC1 activation, allowing the repression of growth promoting signals under hypoxia.

## 4. AKT Signaling in Hypoxic E6/E7 Repression

Notably, the PI3K/AKT/mTOR pathway is not only important for the phenotypic response of hypoxic HPV-positive cancer cells where the hypoxia-linked inhibition of mTORC1 signaling impairs their senescence response [19]. A recent study revealed that the repression of the viral oncogenes in hypoxic HPV-positive cancer cells is dependent on AKT in that interference with AKT signaling counteracts the hypoxic HPV E6/E7 repression [25].

AKT has been reported to be phosphorylated under hypoxic conditions in different tumor cells [21,22], which also include HPV-positive cervical cancer and HPV-positive and -negative head and neck squamous cell carcinoma (HNSCC) cells [23,25]. The exact mechanism of hypoxia-induced activation of AKT in HPV-positive cancer cells is still unclear. Both the HPV E6 and E7 oncogenes have been shown to possess the potential to induce the PI3K/AKT pathway [58,59,60]. However in hypoxic HPV-positive cancer cells, AKT activation is observed under conditions of E6/E7 repression and is even causative for this E6/E7 repression [25]. This indicates that the hypoxic AKT activation is regulated by an E6/E7-independent mechanism. In other cell models, it has been found that the enhanced AKT activation under hypoxia can be the result of a reduced activity of the oxygen-dependent hydroxylase EglN1 that inhibits AKT under normoxia [61]. Whether AKT activation in hypoxic HPV-positive cancer cells is caused by an impaired oxygen-dependent hydroxylation of AKT remains to be investigated. The hypoxic AKT activation and subsequent repression of E6/E7 are dependent on the canonical AKT upstream activators PI3K and mTORC2 (Figure 1), as was shown by applying chemical inhibitors and genetic tools [25]. Thus, in contrast to the impaired mTORC1 signaling, mTORC2 is active under hypoxia in HPV-positive cancer cells. This is in line with only mTORC1, but not mTORC2, being sensitive to fasting and cellular stress such as oxygen deprivation [36]. Moreover, hypoxic repression of mTORC1 signaling may abrogate the mTORC1-mediated feedback inhibition on mTORC2/AKT that can be induced under nutrient- and energy-replete conditions [24].

All three AKT isoforms, AKT1-3, can be activated via the PI3K/mTORC2 cascade [24]. They are characterized by isoform-specific but also by redundant functions. This is demonstrated by the distinct phenotypes observed in isoform-specific knockout mice [62,63,64] and by the viability of single isoform knockout mice in contrast to the lethality of combined knockout mice [65,66]. Isoform-specific expression and functional analyses indicate that AKT1 and AKT2 mediate the hypoxic repression of the HPV E6/E7 oncogenes by acting in a functionally redundant manner [25].

Interestingly, AKT-induced repression of E6/E7 is observed only in hypoxic but not in normoxic HPV-positive cancer cells, indicating the requirement for a hypoxia-adapted cellular background [25]. Moreover, hypoxia-induced AKT activation and E6/E7 repression are glucose-sensitive and counteracted by high glucose concentrations [19,25]. Under mechanistic aspects, it will be interesting to further decipher which factors act downstream of AKT to mediate the hypoxic HPV oncogene repression and which are the determinants that restrict the effect of AKT signaling on E6/E7 expression to hypoxic cells [19,25].

## 5. Role of the PI3K/AKT/mTOR Pathway in HPV-Positive Tumors in the Clinic

An aberrant activation of the PI3K/AKT/mTOR pathway is frequently found in many types of cancer and contributes to their malignant growth and therapy resistance [26]. Alterations in this pathway are often caused by an activating mutation in the PIK3CA gene, which encodes the catalytic subunit of class I PI3K, or by genomic amplifications thereof. However, also the loss of the negative regulator PTEN or changes in the expression or activity of further pathway members such as RTKs or AKT can play a role [28]. Consequently, intense research efforts are focusing on investigating the PI3K/AKT/mTOR pathway as a promising therapeutic target for cancer treatment in clinical studies. However, many of the studied PI3K/AKT/mTOR inhibitors exhibited only modest anticancer activities in patients and/or prohibitive toxicities, and only a small number of these agents have thus far been approved for clinical treatment of a small number of selected cancers [27,67]. As a consequence, much effort is currently concentrating on the development of more potent and more selective PI3K/AKT/mTOR inhibitors with less toxicity, their use in combination with other treatment strategies, and the identification of predictive biomarkers to select for patients which may particularly benefit from the therapeutic targeting of PI3K/AKT/mTOR signaling [27,67].

Similar to various other cancers, the prognosis of cervical cancer patients has been linked to the status of the PI3K/AKT/mTOR pathway. PIK3CA mutations [68,69,70], high levels of phosphorylated AKT [71] and activated mTOR [72,73] were shown to be associated with a worse prognosis and shorter patient survival after chemoradiation. Mutations in PIK3CA are amongst the most frequently detected mutations in cervical cancer [69], but other alterations that result in the aberrant PI3K/AKT/mTOR activation, for instance genomic amplifications of PIK3CA [74,75] or the loss of PTEN [69], are also reported. Moreover, PI3K signaling has been implicated as being important for HPV-induced cellular transformation [76] and HPV oncogenes were found to be able to activate this pathway [58,59,60]. Hence, the PI3K/AKT/mTOR axis is considered to represent a promising therapeutic target also for the treatment of cervical cancer for which the current standard therapy consists of surgery together with radiotherapy or cisplatin-based chemoradiation [2]. Cervical cancer patients are already subjects of (or included in) clinical trials testing PI3K/AKT/mTOR pathway inhibitors (e.g., NCT01958112, NCT01217177, NCT01026792, NCT01226316) [77]. Interestingly, the presence of PIK3CA mutations is associated with better treatment response to PI3K/AKT/mTOR pathway inhibitors, suggesting that this genetic alteration may serve as a marker for personalized medicine in order to predict treatment outcomes for this cancer entity [78,79].

Apart from being closely associated with cervical cancer, HPV also represents a major risk factor for the development of HNSCC, in particular of oropharyngeal cancers (OPCs). Other main risk factors for HNSCC are tobacco and alcohol consumption [80]. The fraction of OPCs attributable to HPV is increasing, in particular in the US and Western Europe where the number of HPV-positive OPCs (70-80%) now exceeds the number of tobacco- or alcohol-linked OPCs [80]. Similar to cervical cancer, the PI3K/AKT/mTOR pathway belongs to the most frequently altered signaling pathways in HNSCC [81]. Notably, mutations in PIK3CA and PTEN are more common in HPV-positive compared to HPV-negative HNSCC [82,83]. Clinical trials in HNSCC patients are therefore evaluating the efficiency of PI3K/AKT/mTOR inhibitors, often using rapamycin analogs [81], and show partially promising treatment responses [84,85].

In consideration of the development of PI3K/AKT/mTOR inhibitors as therapeutics for the treatment of HPV-positive cancer, the close crosstalk of this pathway with the HPV oncogenes may be of clinical relevance. As discussed above, targeting mTORC1 using mTORC1-specific inhibitors (e.g., rapamycin) or dual mTORC1/mTORC2 inhibitors interferes with chemotherapy-induced senescence, but also with senescence induction upon E6/E7 repression [19]. The contradicting contributions to cancer that are attributed to senescent cells, in that they can act both pro- and anti-tumorigenic [86], indicates that the effects of mTORC1 inhibition on tumor growth and treatment response to chemotherapy are complex. The only modest, yet proven, efficacy of mTOR inhibitors for the treatment of various types of cancer [27] may thus be the result of a delicate balance of these effects.

The ability of PI3K/AKT/mTORC2 inhibitors to lead to E6/E7 expression under hypoxia [25] is also noteworthy. This observation may raise concern that the proliferative impulse, which is potentially exerted by E6/E7 may induce the proliferation of malignant cells under hypoxia. However, HPV-positive cancer cells treated with AKT inhibitors under hypoxia do not resume proliferation, at least under in vitro conditions. Further, AKT inhibitors suppress the proliferation of HPV-positive cancer cells under normoxia despite the maintained E6/E7 expression. These findings indicate that the anti-proliferative effect of AKT inhibition is dominant over the growth-promoting stimulus derived from E6/E7 [25].

Since the malignant growth of HPV-positive cancer cells is dependent on the continuous expression of the viral oncogenes, substantial effort is put into the development of E6/E7-targeting therapies [4,87]. Promising potential is attributed to immunotherapy [88], although current approaches are challenged by the ability of HPV to induce immune evasion mechanisms e.g., by modulating the antigen processing machinery or deregulation of the interferon response [89,90]. Additionally, the hypoxic downregulation of viral oncogene expression may support the immune evasion of HPV-positive cancer cells in less oxygenized regions of the tumor [19]. Moreover, hypoxia itself generally acts immunosuppressive and can interfere with immunotherapy [12,13]. On the other hand, PI3K/AKT/mTOR inhibitors have immunomodulatory properties in that they can enhance tumor immune surveillance by reducing the expression of immunosuppressive cytokines and chemokines and by dampening the effector functions of immune regulatory cells like regulatory T cells or myeloid-derived suppressor cells [91,92]. In line with this, PI3K inhibitors have been shown to counteract resistance to immune checkpoint blockade in a mouse tumor model [93] and clinical trials using PI3K inhibitors as a combination therapy together with immune checkpoint inhibitors are ongoing [94,95]. Thus, it will be interesting to explore whether the increase in E6/E7 expression, which is observed under treatment with PI3K/AKT/mTORC2 inhibitors may render hypoxic HPV-positive cancer cells more vulnerable towards E6/E7-targeting immunotherapy.

## 6. Conclusions and Perspectives

**Figure 2:**
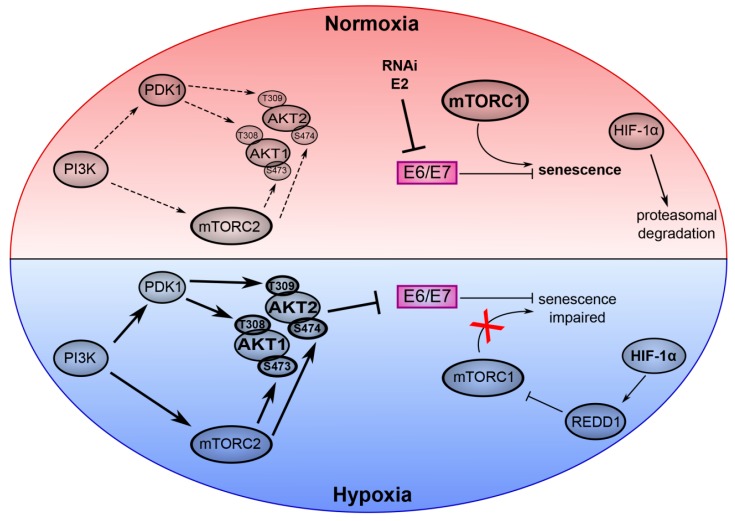
Key role of the PI3K/AKT/mTOR network in the virus/host cell crosstalk in HPV-positive cancer cells under normoxia and hypoxia. The upper part of the model (red) depicts normoxic HPV-positive cancer cells. Here, experimental inhibition of E6/E7 expression by RNA interference (RNAi) or expression of HPV E2 efficiently induces senescence dependent on active mTORC1 signaling. HIF-1α is degraded by the proteasome. The lower part of the model (blue) represents hypoxic HPV-positive cancer cells. Phosphorylation of AKT1 and AKT2 is induced by their canonical upstream regulators PI3K, mTORC2 and PDK1. Hypoxic activation of AKT mediates repression of E6/E7. Hypoxia also leads to the stabilization of HIF-1α, which activates REDD1 and results in the inhibition of mTORC1. Thus, despite the AKT-mediated inhibition of E6/E7, hypoxic HPV-positive cancer cells evade senescence due to impaired mTORC1 signaling. Arrows indicate an activation, bar-headed lines indicate an inhibition. (Copyright © American Society for Microbiology, (mBIO, 10(1), e02323–e02318, 2019 [25]).

Cervical cancer and other HPV-linked cancers are expected to remain a major medical obstacle for the coming decades [4,10,96]. The development of novel treatment strategies is therefore of high clinical importance and a better understanding of the molecular biology of HPV-associated carcinogenesis may provide valuable insights to approach this task. The PI3K/AKT/mTOR signaling cascade plays a key role in the virus/host cell crosstalk of HPV-positive cancer cells. While an activating effect on AKT and mTOR has been attributed to E6 and E7 [58,59,60,97], recent reports highlight that this pathway also has a large impact on E6/E7 expression under hypoxia and on the the phenotypic response of the cell to E6/E7 repression [19,25] (Figure 2).

Signaling via mTORC1 is critical for senescence induction upon E6/E7 inhibition in well-oxygenized HPV-positive cancer cells. However, oxygen deprivation leads to the impairment of mTORC1 signaling, thus, hypoxic HPV-positive cells can evade senescence although E6/E7 is downregulated [19]. Furthermore, the mechanism of E6/E7 repression under hypoxia is also linked to the mTOR signaling circuit since it is mediated by AKT, which is dependent on the activity of its upstream regulators PI3K and mTORC2 [25]. By enabling the efficient downregulation of the viral oncogene expression and simultaneously avoiding the transition into senescence, the interplay of mTORC2/AKT activation and mTORC1 repression is crucial for the induction of dormancy in hypoxic HPV-positive cancer cells. In view of the fact that hypoxic HPV-positive cancer cells pose a major therapeutic problem, it will be an important task for the future to further elucidate how a therapeutic intervention that targets components of the PI3K/AKT/mTOR cascade, could provide the best benefit to patients, in particular considering combinatorial treatments with PI3K pathway inhibitors and E6/E7-targeting (immuno) therapies.
